# Real-Time and Dynamically Consistent Estimation of Muscle Forces Using a Moving Horizon EMG-Marker Tracking Algorithm—Application to Upper Limb Biomechanics

**DOI:** 10.3389/fbioe.2021.642742

**Published:** 2021-02-17

**Authors:** François Bailly, Amedeo Ceglia, Benjamin Michaud, Dominique M. Rouleau, Mickael Begon

**Affiliations:** ^1^Laboratoire de Simulation et de Modélisation du Mouvement, Faculté de Médecine, Université de Montréal, Laval, QC, Canada; ^2^Department of Surgery, Université de Montréal, Montreal, QC, Canada; ^3^Department of Orthopedic Surgery, CIUSSS Nord-de-L'île-de-Montréal, Hôpital du Sacré-Cœur de Montréal (HSCM), Montreal, QC, Canada

**Keywords:** muscle forces estimation, real-time, biofeedback, optimal control, EMG, moving horizon estimation, upper limb

## Abstract

Real-time biofeedback of muscle forces should help clinicians adapt their movement recommendations. Because these forces cannot directly be measured, researchers have developed numerical models and methods informed by electromyography (EMG) and body kinematics to estimate them. Among these methods, static optimization is the most computationally efficient and widely used. However, it suffers from limitation, namely: unrealistic joint torques computation, non-physiological muscle forces estimates and inconsistent for motions inducing co-contraction. Forward approaches, relying on numerical optimal control, address some of these issues, providing dynamically consistent estimates of muscle forces. However, they result in a high computational cost increase, apparently disqualifying them for real-time applications. However, this computational cost can be reduced by combining the implementation of a moving horizon estimation (MHE) and advanced optimization tools. Our objective was to assess the feasibility and accuracy of muscle forces estimation in real-time, using a MHE. To this end, a 4-DoFs arm actuated by 19 Hill-type muscle lines of action was modeled for simulating a set of reference motions, with corresponding EMG signals and markers positions. Excitation- and activation-driven models were tested to assess the effects of model complexity. Four levels of co-contraction, EMG noise and marker noise were simulated, to run the estimator under 64 different conditions, 30 times each. The MHE problem was implemented with three cost functions: EMG-markers tracking (high and low weight on markers) and marker-tracking with least-squared muscle excitations. For the excitation-driven model, a 7-frame MHE was selected as it allowed the estimator to run at 24 Hz (faster than biofeedback standard) while ensuring the lowest RMSE on estimates in noiseless conditions. This corresponds to a 3,500-fold speed improvement in comparison to state-of-the-art equivalent approaches. When adding experimental-like noise to the reference data, estimation error on muscle forces ranged from 1 to 30 N when tracking EMG signals and from 8 to 50 N (highly impacted by the co-contraction level) when muscle excitations were minimized. Statistical analysis was conducted to report significant effects of the problem conditions on the estimates. To conclude, the presented MHE implementation proved to be promising for real-time muscle forces estimation in experimental-like noise conditions, such as in biofeedback applications.

## 1. Introduction

Real-time estimation of muscle forces is a necessary technical breakthrough in biomechanics, as pointed out by various authors (Erdemir et al., 2007; Bouillard et al., 2011). It should help clinicians to determine movements and postures which are likely to cause muscular pain and injuries without compromising diagnosis speed. It is also a promising feature for interactive biofeedback during rehabilitation (Pizzolato et al., 2017b). Muscle force measurements being highly invasive, researchers have turned to musculoskeletal modeling that relies on body kinematics and electromyography (EMG). Some of the algorithms behind these simulations are now fast and mature (inverse/forward kinematics/dynamics) for models without muscles. They have been extensively used for real-time biofeedback of joint angles and moments in rehabilitation applications (Giggins et al., 2013; Richards et al., 2017) or to control active prostheses (Tucker et al., 2015). However, adding muscles to skeletal models widely increases their numerical complexity, requiring either simplifications on the dynamics or very large computational resources. The main hurdle comes from the redundancy of the muscle actuation system which has two consequences: an increase in the number of variables to be controlled/estimated and the need for an optimization layer to resolve this redundancy. The existing approaches for estimating muscle forces can be grouped into three main categories: inverse, forward, and hybrid methods.

Static optimization—an inverse dynamics-based algorithm combined with least-stresses or least-activation criteria has been widely used to estimate muscle forces during various tasks (Crowninshield and Brand, 1981; Raikova and Aladjov, 2002; Heintz and Gutierrez-Farewik, 2007; Morrow et al., 2014). Its computational efficiency has even enabled real-time estimation of muscle forces (Van Den Bogert et al., 2013). However, static optimization presents three main drawbacks: (i) inverse dynamics relies on the double numerical differentiation of noisy kinematic data which results in unrealistic joint torques (Challis and Pain, 2008; Dumas et al., 2016); (ii) the time-independent nature of static optimization prevents the activation dynamics to be accounted for Ackermann and Schiehlen (2009), resulting in non-physiological variations of muscle forces; (iii) the commonly used least-activation criterion makes it unsuitable for retrieving muscle activation of movements or pathologies involving co-contraction (Bélaise et al., 2018a) such as cerebral palsy or stroke (Hu et al., 2013).

Forward approaches, also known as dynamic optimization, address limitations (i) and (ii) by integrating both the equation of motion and the activation dynamics forward in time (Ackermann and Schiehlen, 2009; Bélaise et al., 2018a) while tracking experimental data such as body kinematics or contact forces. Issue (iii) can be tackled by tracking both markers and EMG data as in the so-called “EMG-marker tracking” proposed in Bélaise et al. (2018a) and Moissenet et al. (2019). High computational costs and challenging convergence are the main shortcomings of dynamic optimization approaches which, at first glance, disqualify them for real-time applications. For instance, a 1 s movement with a 6-degree-of-freedom (DoF) arm actuated by 20 Hill-type muscle elements converged in about 60 min with the EMG-markers tracking in Bélaise et al. (2018a). To the best of our knowledge this constitutes a state-of-the-art result.

Finally, hybrid approaches rely on the inverse dynamics to compute reference joint torques which are then tracked using a forward integration of the activation dynamics (Sartori et al., 2012, 2014; Pizzolato et al., 2017a), as seen in the CEINMS toolbox (Pizzolato et al., 2015). These methods address the activation dynamics limitations coming from inverse approaches but maintain the aforementioned problems (i), arising from the double differentiation.

To tackle the computational cost of forward approaches, two strategies can be combined. First, the problem can be implemented as a moving horizon estimation (MHE, Rao et al., 2003). MHE relies on a relatively small subset of past measurements which yields an estimate of the current state via dynamic optimization. Each time this subproblem is solved, the estimation window is shifted forward in time and a new optimization problem is set up, initialized by the previous one, significantly speeding up the convergence. For instance, real-time MHE was used in Bae and Oh (2017) to estimate the simplified state of a humanoid robot or in Quintero et al. (2015) for the coordinated control of two unmanned aerial vehicles. However, to the best of our knowledge, real-time MHE was never applied to a musculoskeletal model in biomechanics. Both the complexity of the system and the size of the estimation window affecting the speed and accuracy of MHE, they need to be jointly investigated in order to satisfy the biofeedback real-time standard ( ≤ 75 ms, Kannape and Blanke, 2013). Second, the solving speed in itself must be improved. To that end, exact derivatives of the optimal control problem (OCP) can be automatically computed thanks to algorithmic differentiation (Andersson et al., 2019), and recent advances in optimal control software such as acados (Verschueren et al., 2019) can be leveraged. In particular, the core of *acados* was written in C (making it remarkably fast) as it is intended to be used on embedded systems, typically for real-time applications.

By feeding a MHE into a fast optimal control software, the main objective of the present study was to assess, for the first time, the feasibility and accuracy of real-time muscle force estimation using EMG and kinematic marker tracking on an arm musculoskeletal model. On an upper limb model, our goals were to satisfy the biofeedback real-time standard and to provide an in-depth insight into the possibilities and limits of the method, paving the way for accurate muscle forces estimation for rehabilitation applications.

## 2. Methods

### 2.1. Musculoskeletal Model

**Figure 1:**
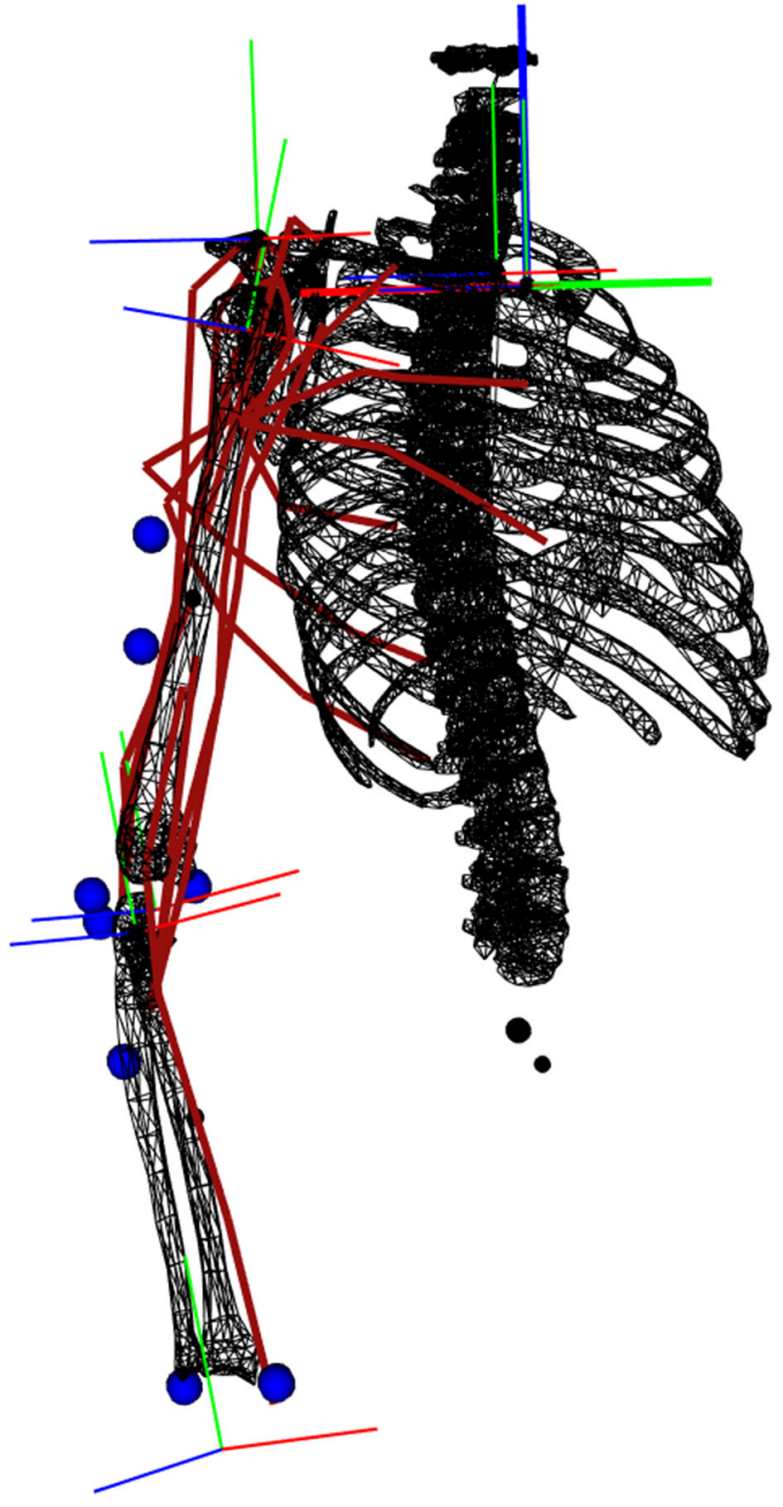
Representation of the musculoskeletal model. The muscle lines of action are depicted in red. The markers are represented by blue spheres. The orthogonal frames are the coordinate systems of each joint.

A 4-DoFs arm actuated by 19 Hill-type muscle elements was developed in the biorbd C++ library (Michaud and Begon, 2021). It consists of three segments: a fixed thorax, an upper arm attached to the thorax with three DoFs in rotation (Glenohumeral plane of elevation, Glenohumeral elevation, Glenohumeral axial rotation) and a forearm attached to the upper arm with one DoF (Elbow flexion), see (Figure 1). Eight markers are placed on the arm, mostly away from large soft tissues: four on Humerus (Medial Deltoid, equal distance between Olecranon and Acromion, Medial and Lateral Condyle), three on Ulna (Olecranon, Ulnar Styloid and lateral side of lower arm) and one on Radius (Radial Styloid). The muscles of the model are: Pectoralis Major (Clavicular, Sternal and Rib parts), Latissimus Dorsi (Thoracic, Lumbar and Iliac parts), Deltoid (Anterior, Middle, and Posterior), Supraspinatus, Infraspinatus, Subscapularis, Triceps (Medial, Lateral, and Long Head), Brachioradialis, Brachial, and Biceps Brachial (Long and Short Head). Lines of action paths are defined using via-points and muscle characteristics are derived from the model of Holzbaur et al. (2005). Two versions of this model were used in this study. In the first one, activation-driven, the controls were the muscle activations and the states were the joint kinematics. In this case, an electromechanical delay of 20 ms was added to EMG data for modeling the time lag between neural excitation and muscle activation (Nordez et al., 2009). In the second one, excitation-driven, the controls were the muscle excitations and the states were the muscle activations and the joint kinematics.

### 2.2. Reference Motion and Physiological Noise

**Figure 2:**
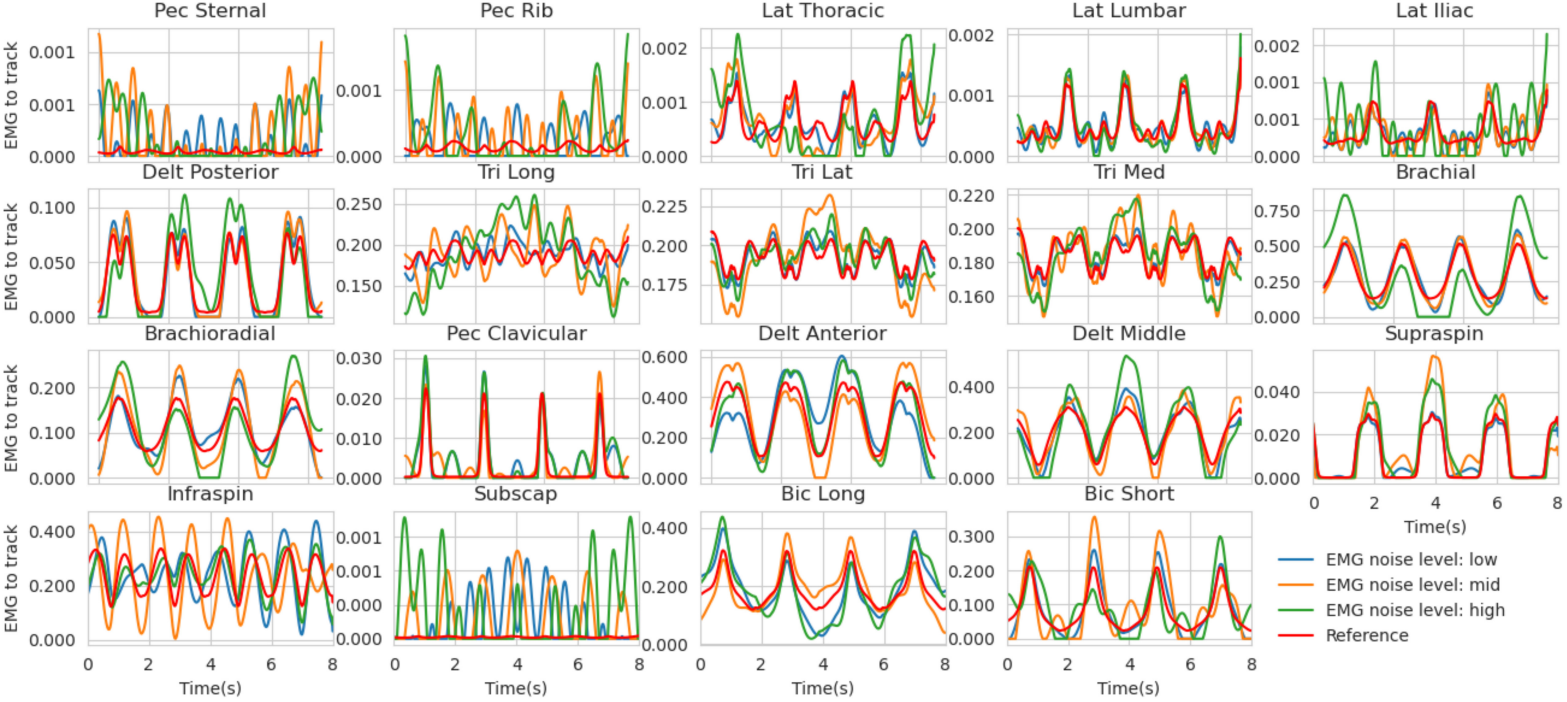
Reference muscle excitations for the mid co-contraction level (red) along with the noised versions (low in blue, mid in orange, and high in red). These are the simulated EMG signals tracked in the estimation problem. Muscle abbreviations stand for (from left to right and top to bottom) : Pectoralis sternal, Pectoralis ribs parts, Latissimus dorsi Thoracic, Lumbar and Iliac parts, Deltoid Posterior, Triceps long head, lateral and medial, Brachial, Brachioradialis, Pectoralis major clavicular, Deltoid Anterior and Middle, Supraspinatus, Infraspinatus, Subscapularis, Biceps Brachial long and short head.

To create reference EMG and kinematic data, a dynamically consistent cyclic motion of the arm was simulated by optimal control. Joint kinematics were imposed at initial and final instants. The control inputs of the model were the muscle excitations, according to Hill's muscle model (Winters, 1990). The objective function to be minimized included weighted Lagrange cost functionals on the controls and on the states (muscle activations and joint angles/velocities). This optimal control problem was solved using a direct multiple shooting formulation with 800 shooting nodes for an 8 s upper-limb motion (corresponding to a 100 Hz data collection). To test the estimator's ability to track several types of excitation profiles, four levels of co-contraction (*Co*_*none*|*low*|*mid*|*high*_) were produced for the same kinematics. They were obtained by adjusting the weights of the cost functions and by targeting minimal levels of excitations (0, 0.1, 0.2, and 0.3) on the muscle elements of the agonist/antagonist pair formed by the Triceps and the Biceps. To estimate the muscle forces in experimental-like conditions, noise was added to the data which were further tracked (EMG, markers). Particular attention was paid to the properties of this artificial noise, to make it as representative as possible of the noise encountered in experimental data. The following manipulations aimed at generating noisy reference data while conserving the ground-truth references for later analysis of the estimator performances:

- EMG - Because of the signal processing pipeline generally applied to processed EMG signals (envelope detection, low-pass filtering), the residual noise should remain in the low-frequency domain (Farina, 2006). To do so, the Fourier transforms of the reference excitations were calculated and its lowest coefficients (up to 2.125 Hz) were randomly biased with four levels of gaussian noise (none, low, mid, high, Figure 2, see details in Supplementary Material 1.). The EMG signals to be tracked were then obtained by inverse Fourier transform.
- Markers - The main errors affecting motion capture markers data come from soft- tissue artifacts and marker placement errors (Gorton et al., 2009; Blache et al., 2017). Therefore, the simulated noise cannot be modeled as a simple additive gaussian distribution on the markers' trajectories. For the following reasons, the soft tissues artifact noise was not modeled: (i) the reference movement of this study is not particularly dynamic; (ii) it comprises very low internal/external rotations; (iii) most of the markers are placed away from large soft tissues. For instance, the Medial Condyle is known for its accuracy (Blache et al., 2017). Therefore, prior to computing reference markers trajectories, placement errors (0, 2, 5, and 10mm, Salvia et al. (2009) ), were simulated by randomly moving the markers on a surrogate model with four levels of centered gaussian noise (none, low, mid and high, respectively). Then, the optimized movement was simulated with this surrogate model in order to obtain altered makers trajectories.

### 2.3. Estimation Problem Formulation

The estimation problem was formulated as a constrained non-linear least-square program, i.e., “to find the controls and the states that minimize the tracking error on the experimental data, while enforcing the dynamics of the system as well as model-related constraints (kinematics, activations, and excitations bounds)”:

(1a) $$\begin{array}{rc} \underset{\begin{array}{c} \underline{x},\underline{u} \end{array}}{\min} & \int_{0}^{T}L\left(\underline{x},\underline{u}\right)dt \end{array}$$

(1b) $$\begin{array}{rcl} \text{s.t.} & \forall t, & \overset{∙}{x}=f\left(x,u\right)\;\text{Dynamics constraints} \end{array}$$

(1c) $$\begin{array}{rc} \forall t, & \underline{u}\in U\;\text{Control path constraints} \end{array}$$

(1d) $$\begin{array}{rc} \forall t, & \underline{x}\in X\;\text{State path constraints} \end{array}$$

(1e) $$\begin{array}{rc} x\left(0\right)=x_{0}, & \text{Initial state constraint} \end{array}$$

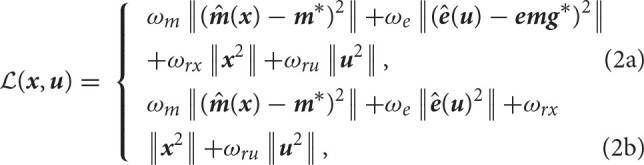


where ***x*** and ***u*** are respectively the state and control vector trajectories. Equation (1b) is the forward dynamics function that implements the ordinary differential equations of the rigid-body and muscle dynamics. Equation (1c) are the control path constraints enforcing muscle excitations in [0, 1]. Equation (1d) are the state path constraints enforcing the kinematics limits of the model ([−π/2;π/2], [−2;π/2], [−π/2;π/2], [−0.5;2.1], in the order of the joints defined in 2.1). Equation (1a) is the running cost to be minimized (detailed in Equations 2 and 2), it includes tracking terms on the marker positions and EMG signals, respectively weighted by ω_*m*_ and ω_*e*_, as well as regularization terms on ***x*** and ***u***, respectively weighted by ω_*rx*_ and ω_*ru*_. ˆ and ^*^ respectively denote estimated and measured quantities. ***m*** represents the marker positions, ***e*** the muscle excitations and ***emg*** the measured emg signals. Three versions of this problem were implemented. In the first and the second versions, the tracked data were the EMG and the markers (Equation 2). They differed in the weights put on the marker tracking (ω_*m*_ = 1e7 or 1e9). In the third version, only the markers were tracked and the squared muscle excitations were minimized (Equation 2), as widely done in the field (Heintz and Gutierrez-Farewik, 2007).

The results provided by all versions were then analyzed and compared (section 2.5). In both approaches, the resulting problems were large constrained non-linear programs which, if treated as a whole, are not solvable in real time and are thus disqualified for biofeedback applications. The OCP was written in Python 3 using bioptim (Michaud et al., 2020), a library dedicated to OCP in biomechanics, interfaced with the cutting edge solver acados (Verschueren et al., 2019). This pipeline also relies on biorbd for all musculoskeletal computations, which were written in CasADi symbolics (Andersson et al., 2019) to take advantage of automatic differentiation and generate the required Jacobians and Hessians for the non-linear solver.

### 2.4. Moving Horizon Estimation of Muscle Forces

A MHE algorithm was implemented to split the estimation problem in a succession of smaller ones. The idea is to process fixed-size subsets of the tracking data moving forward in time. In the following, we refer to this moving horizon as a MHE window. Each time one subproblem is solved, a new measurement is acquired, the oldest one is discarded and a new subproblem is defined, of which only a few numerical values differ from the previous one. Due to this similarity, an efficient warm-starting strategy using the previously estimated state and control trajectories can be implemented to speed up the convergence of the successive problems to be solved. The dynamic consistency of the final solution is enforced by appropriate constraints on the initial state. Convergence was considered achieved when more than 90% of the iterations actually converged. Pseudocode for this algorithm is provided below:

**Algorithm 1:** MHE pseudo-code

1: mhe_nlp ← new_mhe_nlp() // create nonlinear program
2: mhe_nlp.init_data_to_track(sensor_data) // get initial data to track
3: [x, u] ← mhe_nlp.solve() // solve nlp and get state and control trajectories
4: store_estimation(x[0], u[0]) // store first estimates of state and control
5:
6: while true do
7: if new_measurement_available then // move forward in time
8: mhe_nlp.update_data_to_track(sensor_data) // get new measurement and discard old one
9: mhe_nlp.update_initial_constraint(x[1]) // impose first state for dynamic consistency
10: mhe_nlp.warmstart(x, u) // provide nlp with initial guess from previous solve
11: [x,u] ← mhe_nlp.solve() // solve nlp and get state and control trajectories
12: store_estimation(x[0], u[0]) // store first estimates of state and control

### 2.5. Analyses

First, the MHE was applied to noiseless data to assess the soundness of the approach and to study its accuracy with regard to an offline full window estimation (section 3.1). To that end, MHE window sizes varying from 3 to 20 were used and each time both the convergence time and the root mean square errors of the estimates were collected and compared to the ones of the full window approach. As the reference data was generated at 100 Hz, which is faster than the MHE performances, EMG signals and marker positions were appropriately subsampled for each window size, in order to simulate real-time conditions. The MHE was run 100 times per window size to compute its average running frequency. Based on these results, a speed/accuracy tradeoff was chosen by fixing the window size for subsequent analyses.

Then, the MHE was run on data with the physiological noise (section 2.2). To assess the effects of the marker noise (four levels, none, low, mid, and high), of the EMG noise (four levels, none, low, mid, and high) and of the co-contraction (four levels, none, low, mid, and high), a fully crossed experimental design was conducted resulting in 64 experimental conditions, each repeated 30 times. Each time, the root mean square error was calculated for the joint angles, marker positions and muscle forces, with respect to the reference data. The effect of EMG noise and co-contraction on muscle forces estimation was assessed using a two-way analysis of variance and postdoc *t*-test with Bonferroni correction (section 3.3). The effect of marker noise, EMG noise and marker tracking weight on joint angles estimation was assessed using a three-way analysis of variance and *post-hoc t*-tests with Bonferroni correction (section 3.4). All computations were performed on a personal computer (IntelⓇ Core*TM* i7-6700HQ CPU @ 2.60 GHz) with parallelization on eight threads.

## 3. Results

### 3.1. Performance of the MHE on Noiseless Data

**Figure 3:**
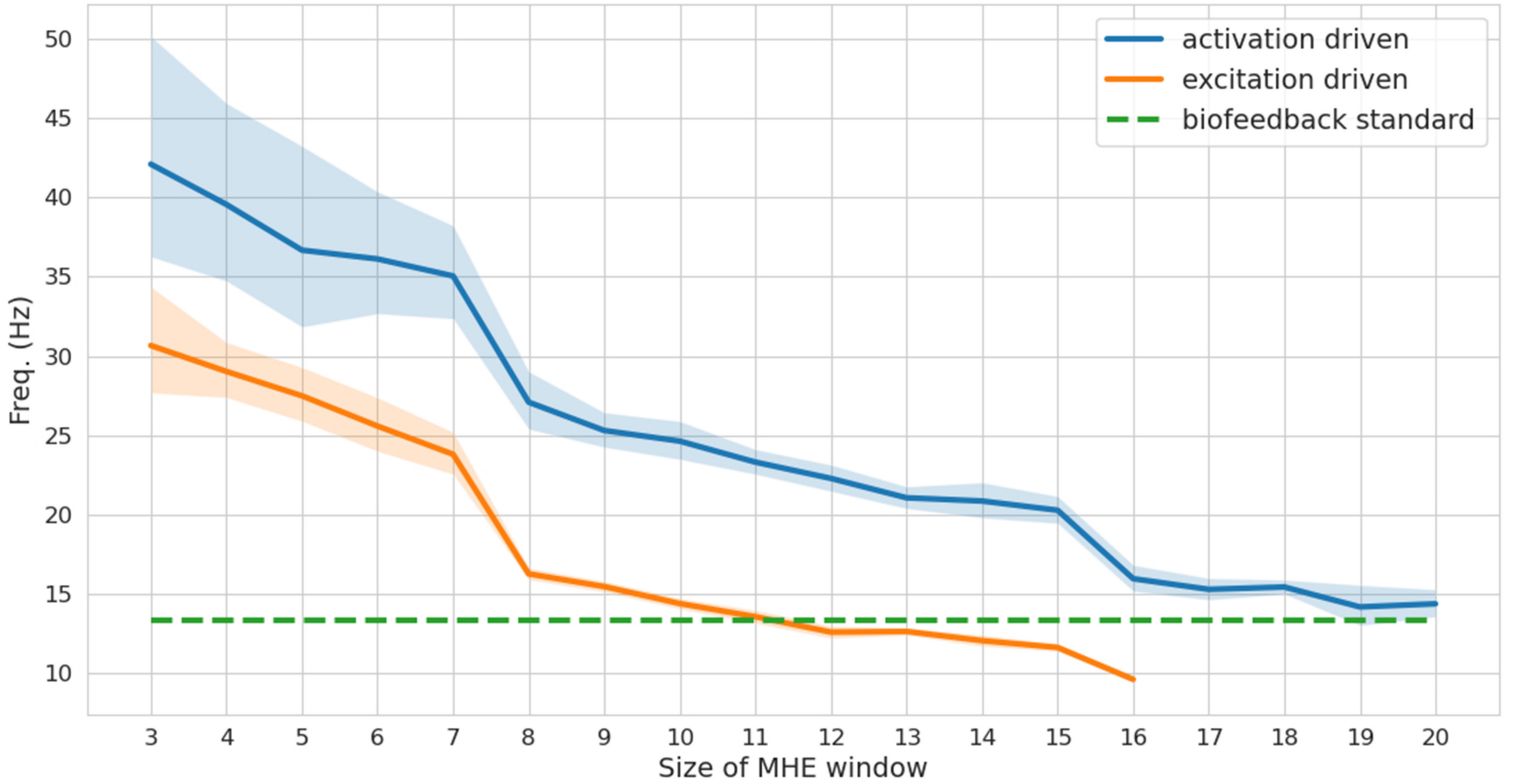
Frequency (mean and standard deviation for 100 tests) at which the moving horizon estimator (MHE) runs as a function of the window size, for activation and excitation driven formulations. The biofeedback standard is depicted in dashed green.

While a full-window estimation is not appropriate for real-time feedback (it is necessarily an offline approach), it is interesting to report its performance in order to better appreciate the improvements brought by the MHE implementation. The full window estimation took 13.86 and 29.80 s for the activation-driven and excitation-driven models, respectively (1.7–3.7 times real-time performances, since the reference motion lasts for 8 s). With the MHE formulation, the computation times were drastically reduced, enabling real-time estimation. The frequency at which the MHE runs decreases as the window size increases (Figure 3), from 30 to 10 Hz for the excitation-driven formulation. Decreasing the complexity of the model, i.e.,, from excitation- to activation-driven, increases the speed of the estimation, by an average of 9.6 Hz. The excitation-driven (respectively activation-driven) formulation was faster than the real-time biofeedback standard (13.3 Hz) up to a window size of 11 (respectively 20).

**Figure 4:**
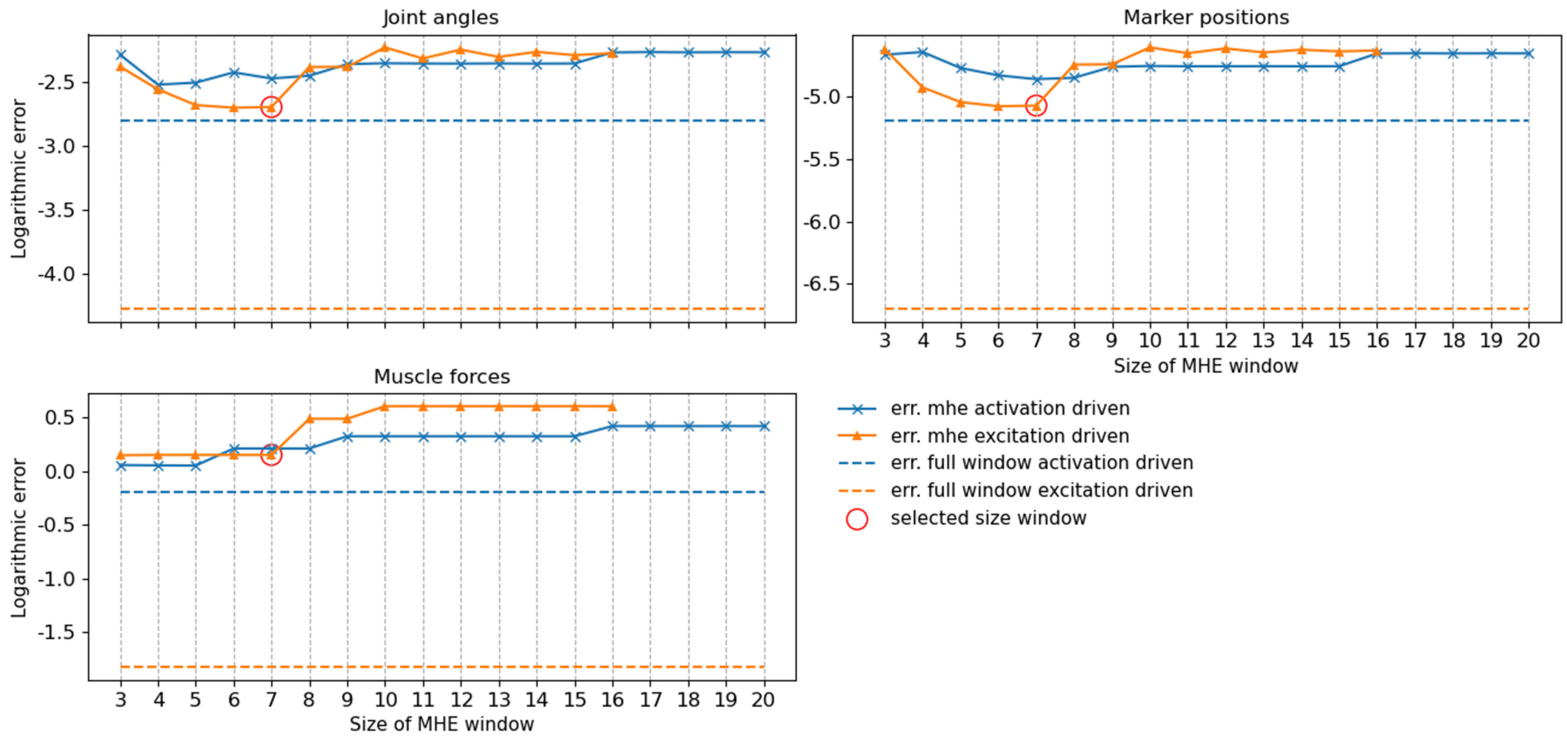
RMSE of the moving horizon estimation (MHE) and full window estimation for joint angles (q, deg), muscle forces (N) and marker positions (m) as a function of the window size, for the excitation-driven and activation-driven formulations. RMSE are displayed in decimal logarithmic scale for the sake of visualization.

The accuracy of the full window estimator and of the MHE for increasing window sizes and for both formulations (activation/excitation-driven) for joint angles, muscle forces and marker positions was computed on noiseless measurements (Figure 4). The estimation error is larger on all components when comparing MHE to full window estimation, which is an expected result given the lesser amount of measurement data available for the MHE. In the excitation-driven formulation, estimation errors on joint and marker positions decrease up to a window size of 7 and then increase with the length of the window size. The estimation error on muscle forces is constant up to a window size of 7 and then increases with the length of the window size. Similar results can be observed for the activation driven formulation. In what follows, results are presented for the excitation-driven formulation. Indeed, we chose to conduct further analysis with the most advanced model to demonstrate its ability to deal with activation dynamics, despite the good performances exhibited by the activation-driven formulation in both speed and accuracy (see section 4 for a discussion). In this regard, a window size of 7 was chosen because it allows the estimator to run around 24 Hz, while ensuring the lowest RMSE on joint kinematics and muscle forces (joint angle errors <0.01, muscle force errors 1 N, marker positions error <0.01 mm).

### 3.2. Muscle Force Estimates in the Presence of Marker and EMG Noise

**Figure 5:**
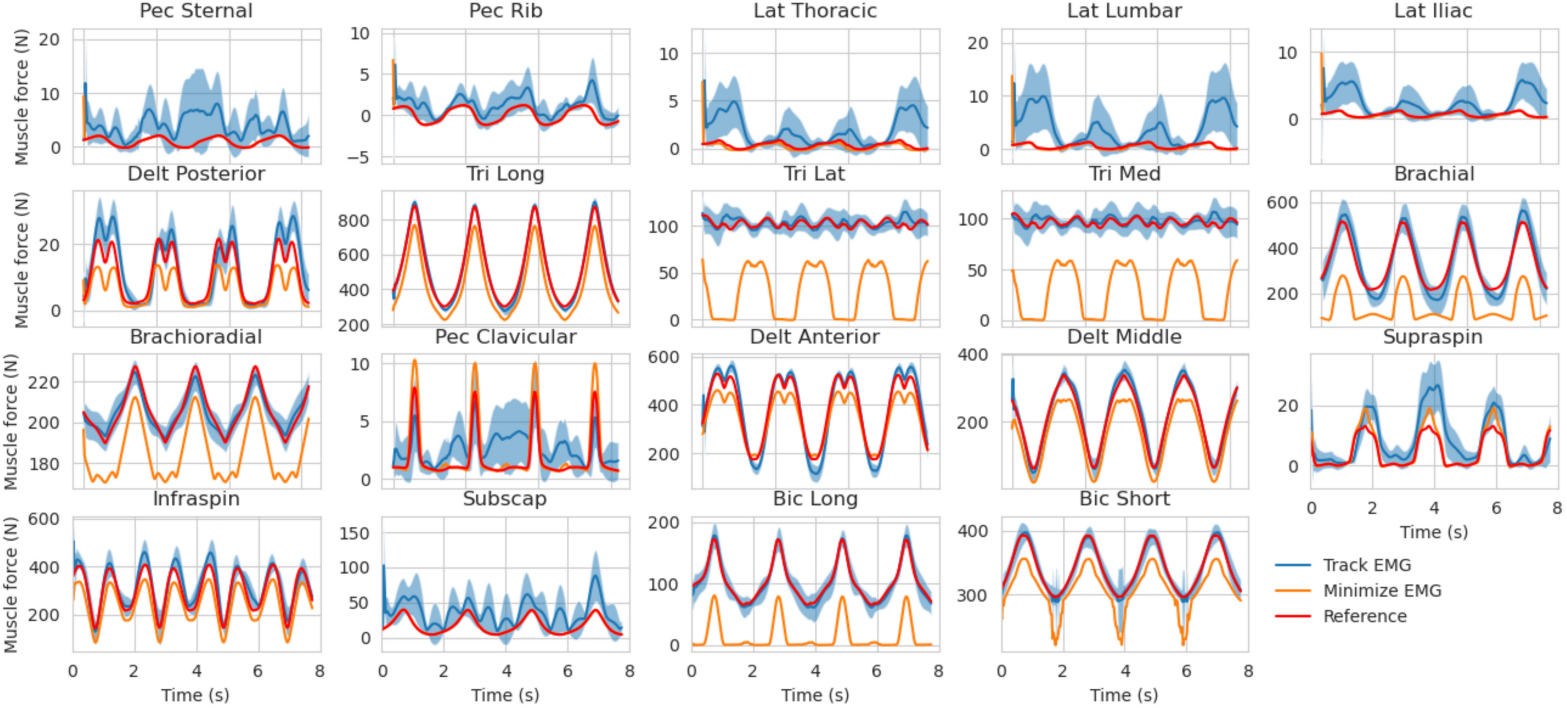
Muscle forces estimation obtained with the MHE algorithm for the mid co-contraction level, mid marker noise level and mid EMG noise level (mean ± std, 30 trials). The muscle force references (from the simulation) are represented (in red) along with the estimates obtained by EMG tracking (in blue) and the estimates obtained by minimizing muscle excitations (in orange).

The muscle forces obtained with our method are consistent with the reference trajectories (Figure 5). When minimizing muscle excitations, the muscle forces are accurate for most muscles, except for the ones where the co-contraction occurs (e.g., Triceps medial and lateral, Brachioradialis, Brachial and Biceps Brachial long and short). The variability of the EMG-tracking solutions (blue, Figure 5) comes from the random noise injected to reference muscle excitations (Figure 2). In the remainder only the trials for which more than 90% of the optimizations have converged will be kept for analysis, the others will be ignored. Across all conditions, the mean convergence rate was 98.95% ranging from 80 to 100%.

### 3.3. Effect of Excitation Noise and Co-contraction on Muscle Forces Estimation

**Figure 6:**
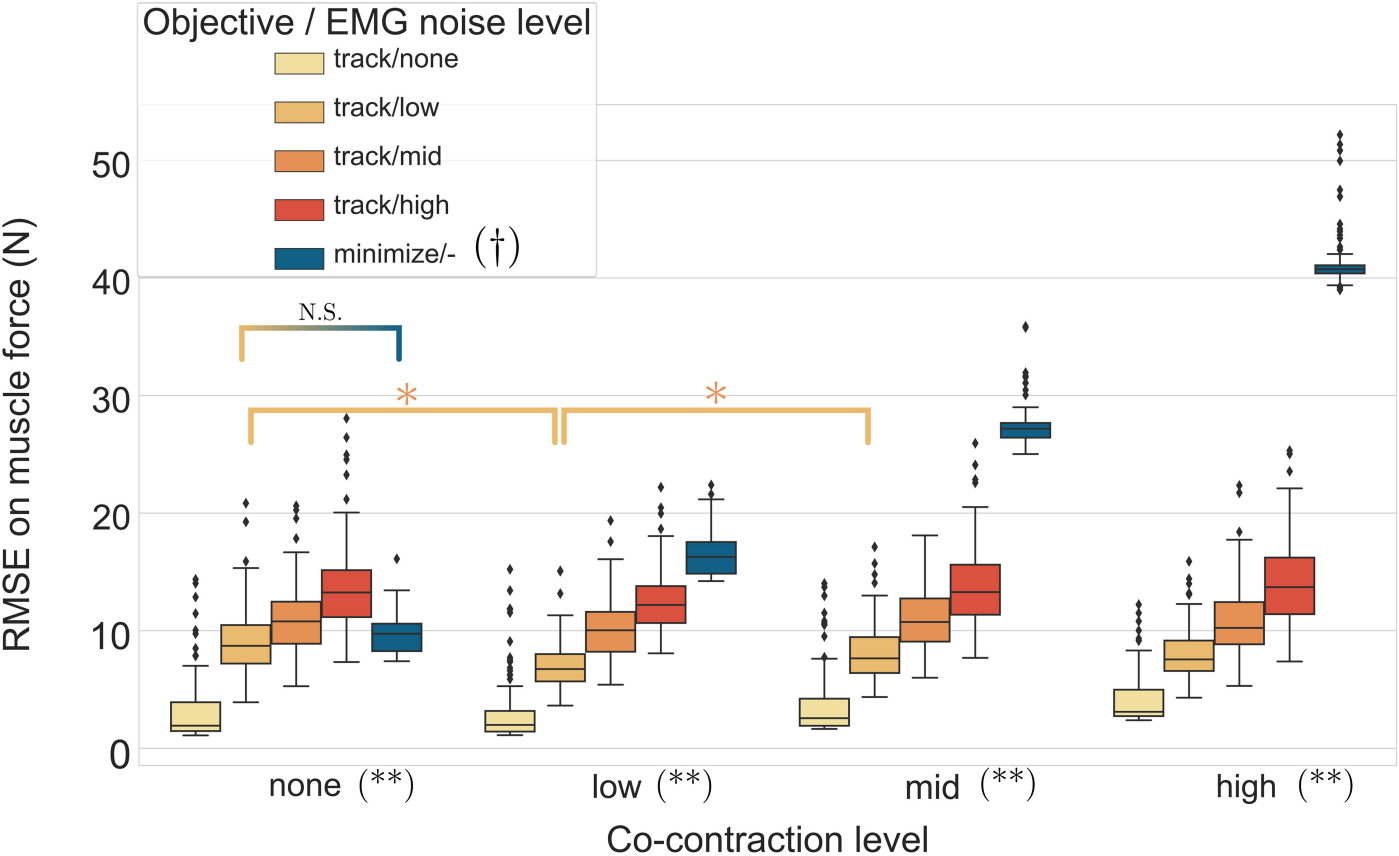
RMSE (30 trials x 4 marker noise conditions per boxplot) of the MHE on muscle forces as a function of the co-contraction level, the EMG noise level (color coded from lvl:none to lvl:high) and the type of objective function (EMG tracking or minimizing excitations). * stands for significantly different RMSE (*p* < 0.001). † stands for significantly different RMSE for one objective type across all co-contraction levels. (**) stands for significantly different RMSE (*p* < 0.001) for all objective types inside one co-contraction level, unless stated otherwise by N.S. Inside each objective type, if not stated otherwise, the RMSE are not significantly different.

The two-way ANOVA presented significant interactions which are summed-up in Figure 6. Inside each co-contraction the RMSE on muscle forces mainly increases with the EMG noise level (except for one test), with the excitation-minimizing formulation (blue boxplot) leading to a bigger RMSE for low, mid, and high co-contraction levels. Moreover the EMG-tracking formulation can track co-contraction (the RMSE of EMG-tracking solutions are not significantly sensitive to the co-contraction level, except for two tests) whereas minimizing the excitations leads to a RMSE increasing with the level of co-contraction (*p* < 0.001).

### 3.4. Effect of Marker Tracking Weights, Marker Noise, and EMG Noise on Joint Angles Estimation

**Figure 7:**
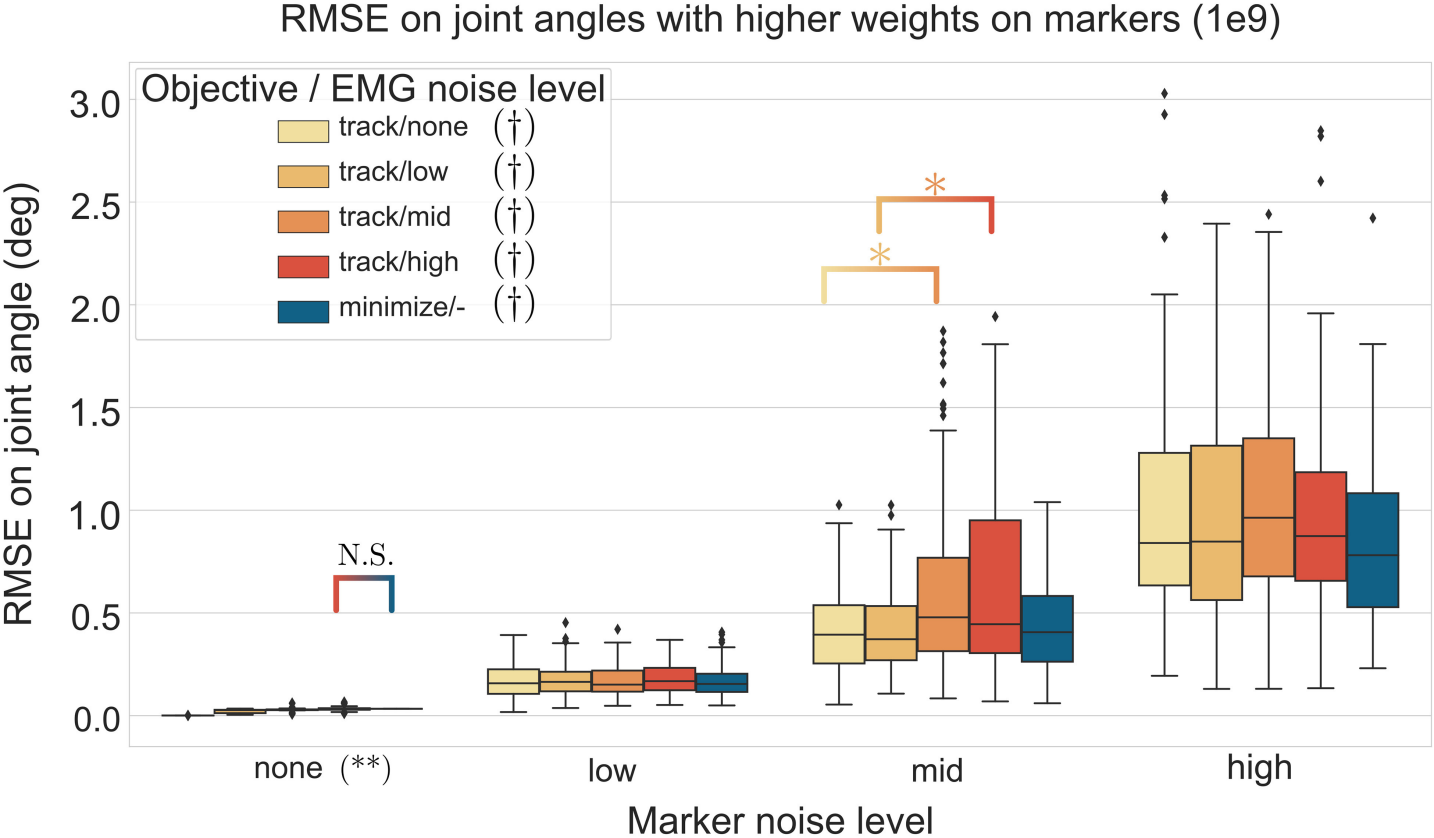
RMSE (30 trials x 4 co-contraction level conditions per boxplot) of the MHE on joint kinematics as a function of the marker and EMG noise levels (color coded from lvl:none to lvl:high) and of the objective function (EMG tracking or minimizing excitations), with a high weight on the marker tracking. * stands for significantly different RMSE (*p* < 0.001). † stands for significantly different RMSE for one objective type across all marker noise levels. (**) stands for significantly different RMSE (*p* < 0.001) for all objective types inside one marker noise level, unless stated otherwise by N.S.

**Figure 8:**
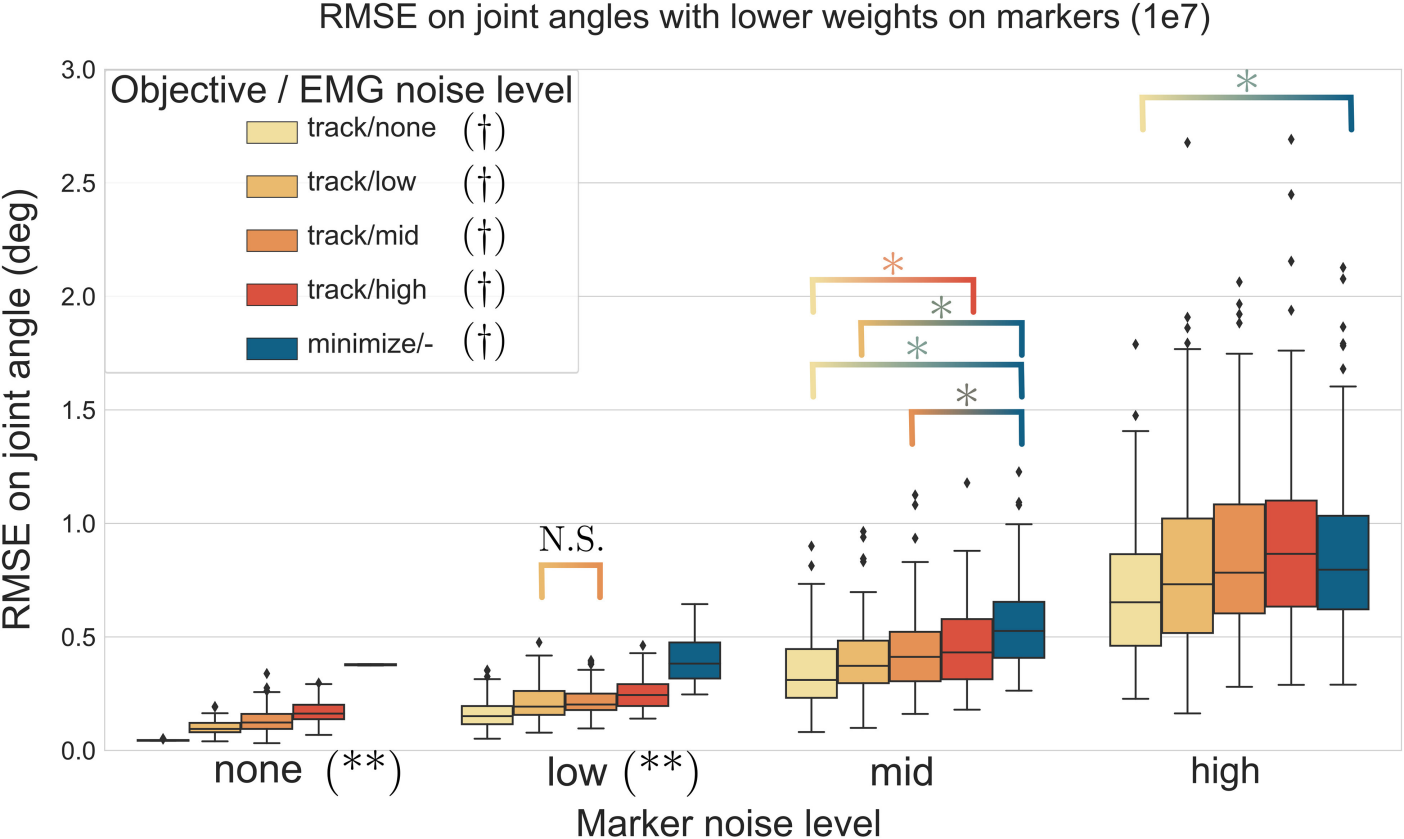
RMSE (30 trials x 4 co-contraction level conditions per boxplot) of the MHE on joint kinematics as a function of the marker and EMG noise levels (color coded from lvl:none to lvl:high) and of the objective function (EMG tracking or minimizing excitations), with a low weight on the marker tracking. *stands for significantly different RMSE (*p* < 0.001). † stands for significantly different RMSE for one objective type across all marker noise levels. (**) stands for significantly different RMSE (*p* < 0.001) for all objective types inside one marker noise level, unless stated otherwise by N.S.

The three-way ANOVA presented significant interactions which are summed-up in Figures 7, 8 where the RMSE on the joint kinematics as a function of the marker and EMG noise levels and of the EMG objective (tracking or minimizing excitations), are depicted for higher and lower weights on the marker tracking, respectively. Across all marker noise levels, the RMSE on the joint kinematics increased as the noise on the markers increased (*p* < 0.001). When emphasizing the marker tracking (higher weight, Figure 7), the level of EMG noise did not have a significant effect on the quality of the joint kinematics estimation for the low, mid and high marker noise levels (except for two tests). In this case, essentially, the choice between minimizing or tracking the excitations does not significantly affect the RMSE on joint kinematics. With a lower marker tracking weight (Figure 7), the RMSE on the joint kinematics also significantly depends on the level of EMG noise (it systematically increases with the level of noise, with the excitation-minimizing formulation leading to a higher RMSE, *p* < 0.001). But in this case, the RMSE on the joint kinematics is always significantly smaller when tracking the EMG signals for the none level and almost always for the low and mid levels, than when minimizing muscle excitations (*p* < 0.001).

## 4. Discussion

Our objective was to achieve a real-time and dynamically consistent estimation of muscle forces and joint kinematics from EMG signals and marker positions. As a proof-of-concept, we used a 4-DoFs and 19 muscle elements arm model. We found that this estimation problem was tractable in real time, estimating muscle forces at up to 30 Hz. To guarantee dynamic consistency, the problem was formulated as a moving-horizon forward approach discretized using a direct multiple shooting formulation.

Compared to state-of-the-art dynamically consistent estimation of muscle forces and joint kinematics (Bélaise et al., 2018a), the tremendous speed increase (3500x real-time vs. real-time) comes from three major methodological improvements. First, all the dynamic computations were written in CasADi symbolics to efficiently and automatically compute the first and second derivatives required by the non-linear solver (Andersson et al., 2019). Moreover, the problem was solved using the fast non-linear solver acados (Verschueren et al., 2019). To the best of our knowledge, this is the first time that acados was used in a biomechanical study. We believe that it is a very promising tool for optimization-in-the-loop biomedical applications with real-time expectations. As shown by the performances of the full window estimation (see section 3.1), the combination of these two contributions resulted in a 945-fold increase (3.7x vs. 3500x real-time) of the resolution speed. Finally, by formulating the estimation problem on a moving horizon, which is a necessary condition to be able to process data on the fly and implement true real-time software, the remaining speed improvement was achieved, without compromising the convergence (98.95% success rate on 1920 different problems). With the prospect of making our code accessible to the largest audience, an effort was put in the development of a Python interface (Michaud et al., 2020), to facilitate the setup and help solve general optimization problems in biomechanics. This should be of particular interest to the community (C++ is used in other available solutions, Bélaise et al., 2018a; Dembia et al., 2019).

First, the estimation algorithm was run onto noiseless measurements to report its behavior with regard to a classic full window approach. This step was essential to choose the size of the estimation window that best met the speed/accuracy tradeoff. Indeed, in section 3.1 it was reported that the RMSE tended to increase with the length of the window size. This is explained by the fact that, as the window size increases, the solving speed decreases, thus new measurements are more distant from one another, the quality of the initial guess is degraded and each subproblem is harder to solve. We also showed that, for the model and motion investigated in this work, the activation-based formulation accuracy was similar to the excitation-based one. This is the result of the activation-based formulation being faster (by about 10 Hz for a same window size), meaning that the tracked measurements are subsampled by a smaller factor and the resulting optimization problem is therefore better informed. Before deciding on the relevance of modeling activation dynamics when estimating muscle forces, this finding should be further investigated on a wider range of models and motions. As stated earlier, to illustrate the ability of our formulation to deal with activation dynamics, the excitation-based formulation was kept for further analyses. For the model used in this study, a 7-sample window appeared to be the optimal choice. It led to the lowest error on all variables of interest, while running at 24 Hz, which is approximately twice the biofeedback standard. This time margin of about 80 ms will be crucial when carrying the method out in an experimental context as it leaves time for other computing tasks while remaining in real-time (e.g., EMG and markers processing, visualization, warm-starting with an extended Kalman filter, etc.). All these side computations are commonly carried out in real-time (Zohar and van den Bogert, 2008; Menegaldo, 2017; Pizzolato et al., 2017b, 2020) and could be run in parallel, opening up the possibility of working with more complex models. For instance, in Pizzolato et al. (2017b), data processing in Vicon Nexus, time delays caused by the filtering phase shift and refresh time of the monitor used to provide the visual biofeedback accounted for 50% of the total processing time, i.e., 58 ms. Since our implementation is parallelized (specifically the integrations of the shooting intervals), the main leverage to increase our solving rate would be the number of cores (unreported results). Forward approaches are also able to handle the fact that in practical situations, it is frequent that only a limited number of muscle EMGs are available for tracking. In that case, several heuristics can be implemented to overcome this lack of information [as done in CEINMS, Pizzolato et al. (2015) ], such as mapping, synergies or least activation criterion for the uninformed muscles.

The performances of the present method should be put in perspective with the static optimization results in Van Den Bogert et al. (2013), where joint kinematics and kinetics of a 44-DoFs full-body model with 300 muscle elements were computed at 120 Hz and of the hybrid approach in Pizzolato et al. (2017b), where joint kinematics and kinetics of a 23-DoFs lower-body model with 34 muscle elements were computed at 17.5 Hz (excluding aforementioned side computations). In terms of number of DoFs and muscle elements, the model used in the present study is simpler. Compared to Van Den Bogert et al. (2013), the estimation rate is also much lower, but promisingly, it is above the one reported in Pizzolato et al. (2017b). As stated in the introduction, the computational burden of our method is the price to pay to estimate dynamically consistent muscle forces and joint kinematics. Given the computational breakthrough reported in the present work, we believe that forward approaches, once disqualified for real-time applications, should be investigated further because they provide physiologically plausible estimates of muscle forces, without the issues surrounding differentiation of ill-conditioned kinematic data. In order to have a sense of how the presented method would scale up to more complex upper limb models such as the ones developed in Saul et al. (2015) and Rajagopal et al. (2016), we conducted a quick test. Sixteen muscles from our original model were duplicated with slightly modified insertion points and isometric forces reduced by half. With this 35 muscle elements model, both formulations performed similarly to the previous model in terms of estimation errors. On a regular laptop, the excitation-driven formulation ran at 10 Hz, which is under the biofeedback standard. The activation-driven formulation however ran at 21 Hz which satisfies the biofeedback standard. On a regular laptop, this would certainly imply to go with the activation-driven formulation which was shown to be comparable to the excitation-driven one on this type of motions. Future improvements in optimal control software and in the computational efficiency of processors should quickly allow the application of this method to bigger models while remaining in real-time.

In a second stage, the MHE algorithm was applied to experimental-like data with simulated noise, whose properties were chosen with care. On this occasion, we introduced an new way of simulating EMG noise, using the properties of the Fourier transform. Results from the noise analysis are as expected, as the RMSE on the variables of interest generally increases with the level of the noise. They also confirm the superiority of an EMG tracking formulation over minimizing excitations when processing motions involving co-contraction (Figure 6), reinforcing the findings of Bélaise et al. (2018a); Moissenet et al. (2019), and Lloyd and Besier (2003). We showed that, because our tracking variables are of a completely different nature (marker positions and EMG signals), they complement each other. For instance, when the experimental noise is higher on marker positions and lower on the muscle excitations, tracking the EMG signals improves the accuracy of the joint angles estimation with regard to just minimizing muscle excitations (Figure 8). Even if such noise conditions are unlikely to happen in experimental circumstances (marker kinematics are generally more accurate than EMG) this result illustrates the strength of our formulation which boils down to a dynamically consistent data fusion from different sensors. In a clinical context, where classical motion capture systems are often too demanding to be used, such a method could still give relevant results with less accurate kinematic data (Xsens, Leap Motion), enhanced by EMG signals. In the present work, we chose to work with markers according to the recommendations from Bélaise et al. (2018b), but a recent study suggests that tracking joint angles instead of markers improves convergence (Febrer-Nafría et al., 2020). In terms of solving speed, the outcomes of such a change in the tracked data should be investigated further since, if it simplifies the kinematic tracking term, turning the non-linear quadratic program into a linear one, it requires a round of inverse kinematics. Finally our MHE formulation proved to be a promising solution for estimating muscle forces, even on data with experimental-like levels of noise.

While low-frequency noise was added to EMG signals and although marker positions were biased on a surrogate model, the main limitation of this study is that muscle models (geometry and properties of Hill actuators) and inertial parameters were kept unchanged between the reference and the experimental models, like in Bélaise et al. (2018b); Moissenet et al. (2019). As the MHE is a novel approach in biomechanics, it should be investigated further to quantify its ability to cope with this type of model noise. The estimation error coming from biased inertial parameters should be moderate, especially for slow movements such as the one investigated in the present study. However, an off-line calibration of muscle parameters will be essential before the use of MHE in experimental conditions as done for hybrid approaches in Pizzolato et al. (2017b). Besides, the modeling of the markers positioning error could be improved by enforcing their random displacements on a manifold locally matching the skin surface. Furthermore, we only implemented a single dynamics (without contact forces) as a proof-of-concept. Additional developments (similarly to the changes implemented in Moissenet et al. (2019) compared to Bélaise et al. (2018b)) will therefore be required to adapt the MHE to walking for example, namely the tracking of the contact forces and the transition of the sliding window between phases (implying a change in the set of ordinary differential equations). Moreover, changing the model and the dynamics of the motion might affect the convex speed accuracy trade-off found in this study.

In conclusion, this work demonstrates the relevance of moving horizon forward approaches for muscle forces estimation in biomechanics. Thanks to an appropriate formulation and efficient numerical software, the results show that real-time estimation of muscle forces is achievable on a standard personal laptop. Further studies need to be conducted in order to generalize these findings on several models and motions, but the presented developments are really promising for real-time biofeedback in the context of rehabilitation.

## Data Availability Statement

The datasets generated for this study can be found in online repositories. The names of the repository/repositories and accession number(s) can be found below: https://github.com/fbailly/code_paper_FBB/tree/v1.0.

## Author Contributions

FB and MB: conceptualization and funding acquisition. FB and AC: data curation. FB, AC, BM, and MB: formal analysis and investigation. FB, DR, AC, and MB: methodology. FB, BM, and AC: software. DR and MB: supervision. FB, AC, and MB: writing-original draft. All authors have read and agreed to the published version of the manuscript.

## Conflict of Interest

The authors declare that the research was conducted in the absence of any commercial or financial relationships that could be construed as a potential conflict of interest.
