# Supplementary material for: Real-Time and Dynamically Consistent Estimation of Muscle Forces Using a Moving Horizon EMG-Marker Tracking Algorithm—Application to Upper Limb Biomechanics
Source: Front Bioeng Biotechnol. 2021 Feb 17;9:642742. doi: 10.3389/fbioe.2021.642742 (PMC7928053; doi:10.3389/fbioe.2021.642742)
Supplement: Supplementary file 2 [file Data_Sheet_1.PDF]

# Supplementary Material

## 1 EMG NOISE SIMULATION

In order to simulate experimental noise of EMG signals, the lowest coefficients of the Fourier transform of the reference excitations were biased by addition of independent Gaussian random variables of mean $m$ and standard deviation $s$. For the $i^{th}$ coefficient (with $1 < i < 17$, $i = 0$ corresponding to the zero-frequency coefficient) whose real value is denoted by $FFT_r[i]$, and for the $k^{th}$ level of noise ($0 \leq k < 4$), the mean and std of the noise distribution are:

$$m = \frac{1}{i} FFT_r[i] * n_{lvl}[k], \tag{S1}$$

$$s = 2.5 * FFT_r[i] * n_{lvl}[k], \tag{S2}$$

with $n_{lvl} = [0, 2, 3, 4]^T$, the vector of noise level. The 1/i decay in Eq. (S1) reduces the power of noise as the frequency increases, in line with the low-pass filtering procedures used in the EMG post-processing.

## 2 COMPUTATION OF THE ESTIMATION ERROR

The mean RMSE displayed in Fig. 4 are averaged across all markers, muscles and joint angles. Let us denote by $q_n(k)$, the angle of the $n^{th}$ of N joints at the $k^{th}$ of K shooting nodes. The mean RMSE on joint angles was computed as follows:

$$RMSE_q = \frac{1}{N} \sum_{n=0}^{N} \sqrt{\frac{1}{K} \sum_{k=0}^{K} (\hat{q}_n(k) - q_n^*(k))^2}, \tag{S3}$$

where $\hat{}$ and $^*$ denote the estimated and the ground truth values respectively. In the same way, let us denote by $f_m(k)$, the force of the $m^{th}$ of M muscles at the $k^{th}$ of K shooting. The mean RMSE on muscle forces was computed as follows:

$$RMSE_f = \frac{1}{M} \sum_{m=0}^{M} \sqrt{\frac{1}{K} \sum_{k=0}^{K} (\hat{f}_m(k) - f_m^*(k))^2}, \tag{S4}$$

Similarly, let us denote by $\mathbf{m_l(k)}$, the position of the $l^{th}$ of L markers at the $k^{th}$ of K shooting. The mean RMSE on kinematic markers was computed as follows:

$$RMSE_m = \frac{1}{L} \sum_{l=0}^{L} \sqrt{\frac{1}{K} \sum_{k=0}^{K} (\|\hat{\mathbf{m}}_\mathbf{l}(k) - \mathbf{m}_\mathbf{l}^*(k))\|^2)}, \tag{S5}$$
